# The Prognostic Implication of Late Gadolinium Enhancement Quantification and Syncope in Hypertrophic Cardiomyopathy

**DOI:** 10.3390/jcm14051781

**Published:** 2025-03-06

**Authors:** Christopher Mann, Theresa M. Dachs, Diana Gharib, Katalin Widmann, Rodi Tosun, Marc Srdits, Christina Kronberger, Dietrich Beitzke, Christian Loewe, Andreas A. Kammerlander, Marianne Gwechenberger, Irene M. Lang, Christian Hengstenberg, Thomas A. Zelniker, Daniel Dalos

**Affiliations:** 1 Division of Cardiology, Department of Medicine II, Medical University of Vienna, 1090 Vienna, Austria; christopher.mann@meduniwien.ac.at (C.M.);; 2 Division of Cardiovascular and Interventional Radiology, Department of Biomedical Imaging and Image-Guided Therapy, Medical University of Vienna, 1090 Vienna, Austria

**Keywords:** hypertrophic cardiomyopathy, risk stratification, myocardial fibrosis, magnetic resonance, syncope, ventricular arrhythmia

## Abstract

**Background:** Risk stratification for sudden cardiac death in hypertrophic cardiomyopathy (HCM) remains challenging. Late gadolinium enhancement (LGE) on cardiac MRI signifies myocardial fibrosis and is linked to adverse outcomes in HCM. However, the threshold of LGE that is clinically significant remains a subject of debate. We hypothesized that even small amounts of LGE (≥ 5%) or a history of syncope are associated with worse outcomes. **Methods**: Between May 2018 and June 2023, HCM patients were prospectively enrolled at the Medical University of Vienna, Austria, a tertiary referral center. The primary endpoint was a composite of new-onset ventricular tachycardia, appropriate ICD therapy, and all-cause mortality. **Results**: In total, 230 patients were included. The median age of patients was 56 (IQR 44, 64) years, 40% (*n* = 94) were female, and 43% (*n* = 84) had significant left ventricular outflow tract obstruction (LVOTO). Over a median follow-up of 3.2 years, 29 patients (13%) met the composite endpoint. While the ESC HCM risk score was not associated with the primary endpoint, both LGE > 5% (Adj. HR 6.16) and a history of at least one syncope (Adj. HR 3.40) were independently associated with the primary endpoint. These associations were consistent across patients with and without LVOTO. **Conclusions:** In conclusion, our findings indicate that the combination of a history of syncope together with small amounts of LGE (≥ 5%) in cardiac MRI are associated with unfavorable clinical outcomes in HCM patients

## 1. Introduction

Hypertrophic cardiomyopathy (HCM) is one of the most common heritable cardiac disorders, presenting with a wide variety of anatomical and clinical manifestations, with an estimated prevalence of 1 in 500 individuals [1,2]. Its hallmark is the thickening of the myocardium, especially of the left ventricle (LV), in the absence of any other systemic or cardiac disease [2]. HCM is the most common cause of sudden cardiac death (SCD) in young people, especially in competitive athletes, mainly due to fatal ventricular arrhythmias. Over the past decades, emerging improvements in imaging modalities and genetic testing have enhanced the understanding of this disease's complexity. Although there are several models for estimating the risk of SCD in this population, there is an unmet need for more clinical variables to perform risk stratification on an individualized patient level [3,4].

**Figure 1:**
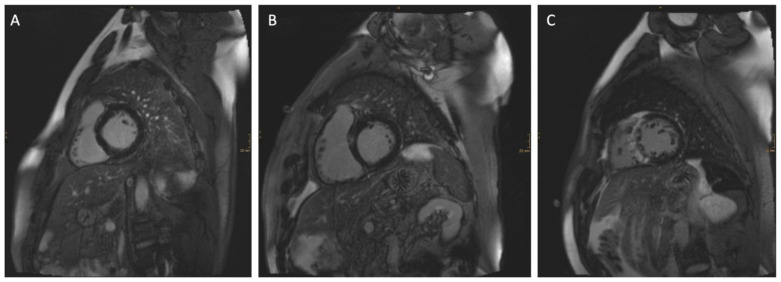
Different amounts of late gadolinium enhancement (LGE) with (**A**) no LGE, (**B**) only traces (< 5%) of LGE, and (**C**) massive (> 15%) LGE.

Cardiac magnetic resonance imaging (CMR) has emerged in the last few years as a useful tool for the diagnosis and differentiation of different forms of HCM. Furthermore, late gadolinium enhancement (LGE) derived by CMR (Figure 1) has emerged as a critical marker of myocardial scar tissue and fibrosis. Several studies have revealed the potential of LGE as a predictor of adverse outcomes in HCM patients [5,6]. Moreover, the current cardiomyopathy guidelines from the European Society of Cardiology (ESC) emphasize LGE above 15% of the LV mass as an additional decision tool in low-risk patients, according to the ESC 5-year SCD risk calculator [3]. However, an ongoing debate discusses the appropriate threshold to delineate those at higher risk [7,8], and a recent study by Greulich and colleagues [9] suggested that a substantially lower threshold may serve as an important predictor of SCD.

Based on this pivotal study and on our clinical experience regarding syncopal events, we hypothesized that a lower LGE threshold of 5% and/or a history of syncope would provide a better prognostic power compared with the established ESC HCM risk score.

## 2. Materials and Methods

### 2.1. Study Design

This observational study was part of the prospective HCM registry at the Department of Medicine II, Division of Cardiology at the Medical University of Vienna, Austria, a tertiary referral center for HCM patients. The study was approved by the local ethics committee (EK1278/2018) in compliance with the Declaration of Helsinki on 22 June 2018. All participants provided written informed consent prior to inclusion.

### 2.2. Patient Population

Between May 2018 and June 2023, consecutive HCM patients were included. Eligibility criteria included confirmed HCM diagnosis, the absence of any ventricular tachycardia (VT) in the medical history, and a complete baseline clinical and imaging dataset. Patients with prior septal reduction therapies or different entities mimicking HCM, such as cardiac amyloidosis, were excluded. Baseline evaluation consisted of a detailed medical history assessment, physical examination, 12-lead electrocardiography (ECG) at rest, ambulatory 48-h Holter-ECG, laboratory assessment, transthoracic echocardiography (TTE), and CMR. Syncope was defined according to the 2018 guidelines of the ESC for the diagnosis and management of syncope, with only episodes deemed likely to be of cardiac origin (e.g., unexplained exertional syncope or syncope associated with arrhythmia) included in the analysis [10]. 

### 2.3. Transthoracic Echocardiography

TTE was performed following standard guidelines using GE Vivid E95 and Vivid E9 machines (GE Healthcare, Wauwatosa, WI, USA). Postprocessing analyses were performed using EchoPAC software V203 (GE Healthcare). All measurements were performed according to current guidelines [11].

### 2.4. Cardiac Magnetic Resonance Imaging

CMR was performed on a 1.5 Tesla scanner (MAGNETOM Avanto Fit, Siemens Healthineers, Erlangen, Germany) following a standardized protocol that has been previously described [12].

LGE was assessed following the administration of 0.15 mmol/kg gadobutrol (Gadovist, Bayer Vital GmbH, Leverkusen, Germany), given that the estimated glomerular filtration rate was > 30 mL/min/1.73 m^2^. The extent of myocardial fibrosis detected by LGE was measured as a percentage of the LV mass using the full width at half-maximum method, as is standard in our center, in a dedicated software by C.K., who was blinded to clinical parameters of all patients (Qmass, Medis Suite MR, Medis Medical Imaging, Leiden, The Netherlands).

### 2.5. Follow up

Patients were consecutively followed every 12 months. Follow-up examinations consisted of medical history assessment, ambulatory 48-h Holter-ECG, and TTE examination, and, if present, implantable cardioverter defibrillator (ICD) device interrogation. 

### 2.6. Study Endpoints

The primary endpoint was a composite of new-onset VT, appropriate ICD therapy, and all-cause mortality. Secondary endpoints were the individual components of the combined endpoint. New-onset VT was defined as ≥ 3 consecutive ventricular beats at ≥ 120 beats per minute. Appropriate ICD therapy was defined as anti-tachycardia pacing and/or shock delivery. The date and underlying cause of death were collected from the national death registry.

### 2.7. Statistical Analysis

Baseline characteristics were summarized as median (IQR) for continuous variables and numbers (percentage) for categorical variables. Differences between groups were compared using the Mann–Whitney U test for continuous variables and the Chi-square test for categorical variables. Kaplan–Meier (KM) event rates were compared using the log-rank test. We used Cox regression models adjusted for age, sex, LV outflow tract (LVOT) gradient, and interventricular septum thickness to assess the relationships between LGE, syncope, and the outcomes of interest. The results are presented as hazard ratio (HR) and 95% confidence interval (CI). A Receiver Operating Characteristic (ROC) curve was used to assess the discriminatory performance of LGE and the presence of syncope. The optimal cut-off point was identified using Youden's index [13]. Differences in the area under the curve (AUC) were tested using the DeLong test. A *p*-value of < 0.05 was considered statistically significant. All statistical analyses were performed using R (version 4.2.2; R Foundation for Statistical Computing, Vienna, Austria).

## 3. Results

In total, 230 patients were included in the study, with a median age of 56 years (IQR: 46–64), and 94 were female (40%). Among these, 84 patients (43%) had significant LVOT obstruction, and the median thickness of the interventricular septum was 19 mm (IQR: 17–22). LGE was present in 68 patients (54%), with a median LGE percentage of 1.8% (IQR: 0–5.4). Additionally, 45 patients (20%) had experienced at least one syncope prior to their baseline visit. During a median follow-up of 3.2 years, 29 patients (13%) met the composite endpoint. Nineteen (8%) had new-onset VTs, four (2%) had an appropriate ICD therapy, and ten (4%) died. Patients in the event group had more often a history of prior syncope (*p* < 0.001) and had a higher use of diuretics compared with event-free patients (*p* = 0.032). The ESC-HCM-SCD risk score did not differ between the two groups. The remaining baseline characteristics are shown in Table 1. Several imaging parameters derived from TTE and CMR were comparable, except for the extent of LGE, which was more pronounced in the event group (9.0% vs. 1.1%, *p* = 0.012, Table 2).

In addition, we observed a significant correlation between the extent of LGE and serum troponin T levels (r^2^ = 0.41, *p* < 0.0001). 

### Association of LGE and Syncope with the Primary Endpoint

**Figure 2:**
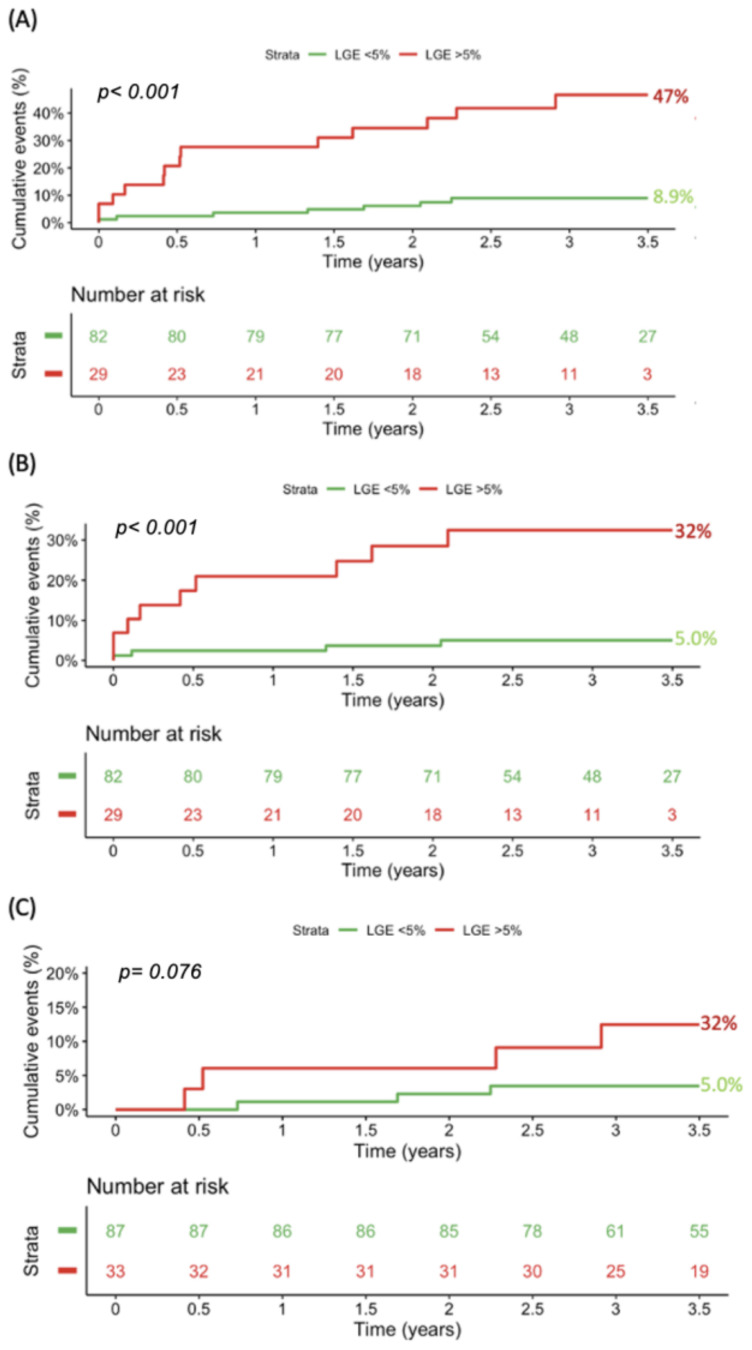
Kaplan–Meier curves and the corresponding event rates for (**A**) the composite of new-onset VT, appropriate ICD therapy, and all-cause mortality; (**B**) new-onset VT; and (**C**) appropriate ICD therapy and all-cause mortality stratified by LGE cut-off of 5%. ICD, implantable cardioverter defibrillator; LGE, late gadolinium enhancement; VT, ventricular tachycardia.

**Figure 3:**
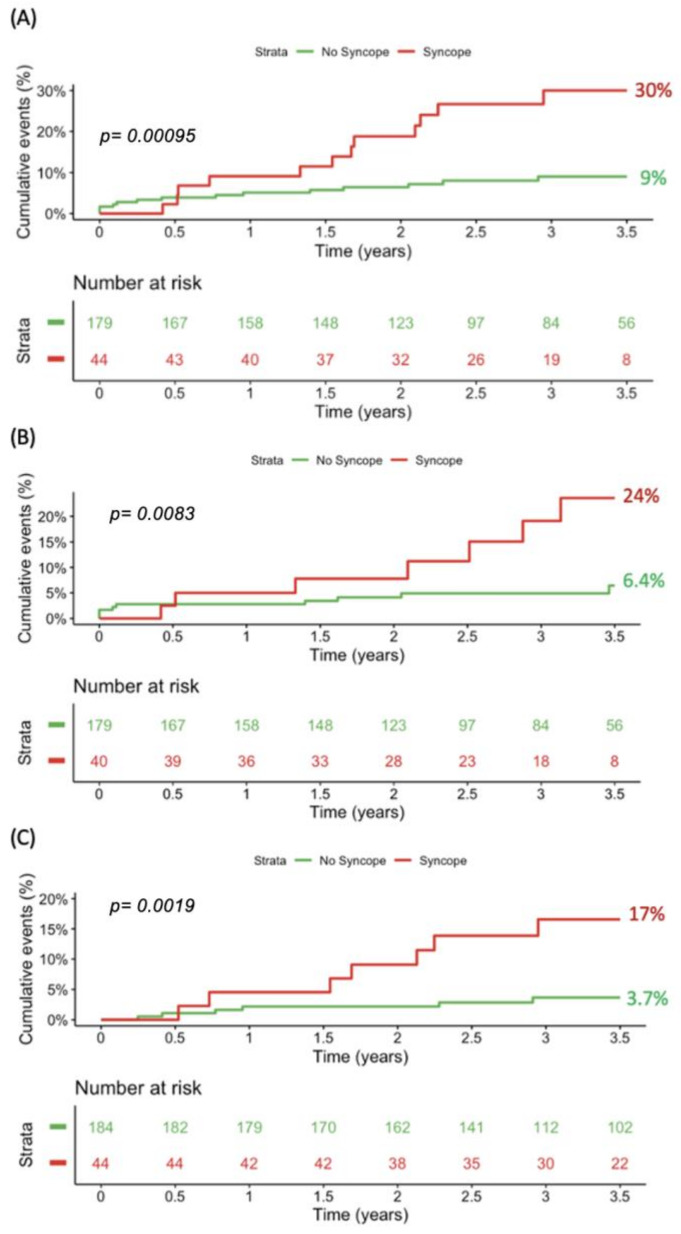
Kaplan–Meier curves and the corresponding event rates for (**A**) the composite of new-onset VT, appropriate ICD therapy, and all-cause mortality; (**B**) new-onset VT; and (**C**) appropriate ICD therapy and all-cause mortality stratified by the presence of syncope. ICD, implantable cardioverter defibrillator; VT, ventricular tachycardia.

Compared with patients with LGE below 5%, those with LGE of 5% and above had significantly higher rates of the primary endpoint (47% vs. 8.9%, *p*-log-rank < 0.0001; Figure 2A). Concerning the secondary endpoints, similar observations were found for new-onset VTs (32% vs. 5.0%, *p*-log-rank < 0.0001; Figure 2B). Although marginally non-significant, a trend was observed for appropriate ICD therapy and all-cause mortality (12% vs. 3.5%, *p*-log-rank = 0.076, Figure 3C).

Patients with at least one syncope prior to the baseline visit had a significantly higher rate of the primary endpoint than those without (30% vs. 9%, *p*-log-rank < 0.001; Figure 3A). Similar results were observed for the secondary endpoints (new-onset VT: 24% vs. 6.4%, *p*-log-rank = 0.008; appropriate ICD therapy and all-cause mortality: 17% vs.3.7%, *p*-log-rank = 0.002, Figure 3B,C) 

**Figure 4:**
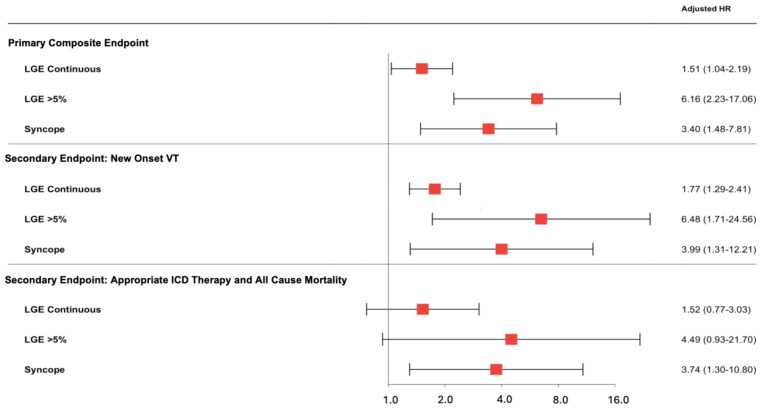
Relationship between LGE as a continuous variable, LGE cut-off of 5%, syncope, and outcomes. Cox regression models were adjusted for age, sex, creatinine, diabetes, heart failure, coronary artery disease, hypertension, atrial fibrillation, LVOT gradient, and interventricular septum thickness. ICD, implantable cardioverter defibrillator; LGE, late gadolinium enhancement; LVOT, left ventricular outflow tract; VT, ventricular tachycardia.

After multivariable adjustment, LGE as a continuous variable was significantly associated with the primary endpoint (Adj. HR for a 1-SD increase in LGE was 1.52 (95%CI 1.04–2.19), Figure 4; Adj. HR for 1% increase was 1.05 (95%CI 1.004–1.11)). Furthermore, an LGE level exceeding 5% and the presence of at least one syncopal event were independently associated with adverse outcomes (Adj. HR 6.16 (95% CI 2.23–17.06), Adj. HR 3.40 (95%CI 1.48–7.81), respectively, Figure 4). 

**Figure 5:**
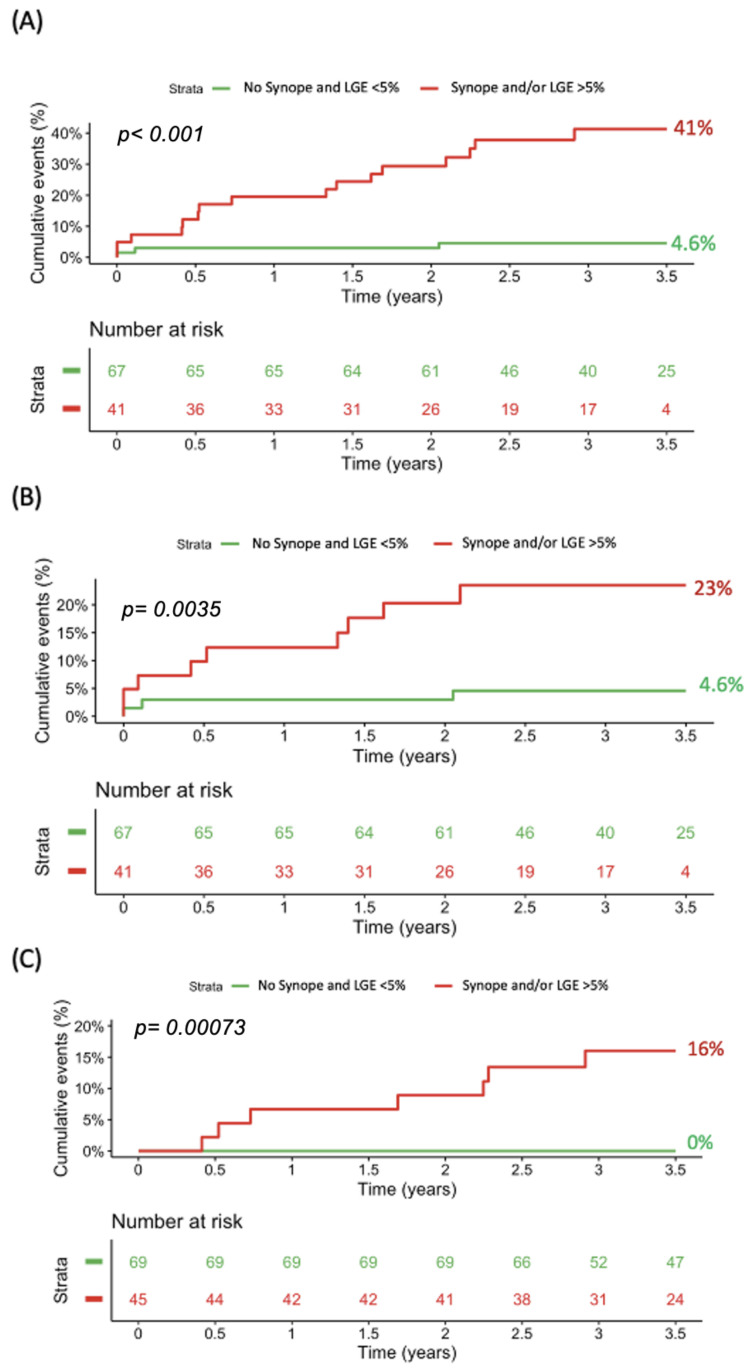
Kaplan–Meier curves and the corresponding event rates for (**A**) the composite of new-onset VT, appropriate ICD therapy, and all-cause mortality, (**B**) new-onset VT, and (**C**) appropriate ICD therapy and all-cause mortality by the presence of syncope and by LGE cut-off of 5%. ICD, implantable cardioverter defibrillator; LGE, late gadolinium enhancement; VT, ventricular tachycardia.

In patients free of both LGE levels exceeding 5% and syncopal events, the primary endpoint event rate was significantly lower than in patients with at least one of these risk factors (4.6% vs. 41%, *p*-log-rank < 0.0001; Figure 5A). Similar results were found regarding the secondary endpoint of new-onset VT (4.6% vs. 23%, *p*-log-rank = 0.003; Figure 5B). Of note, within the cohort with LGE below 5% and the absence of syncope, we did not observe any secondary endpoints of appropriate ICD therapy and mortality compared with 16% of patients with at least one risk factor (*p*-log-rank < 0.001; Figure 5C).

**Figure A1:**
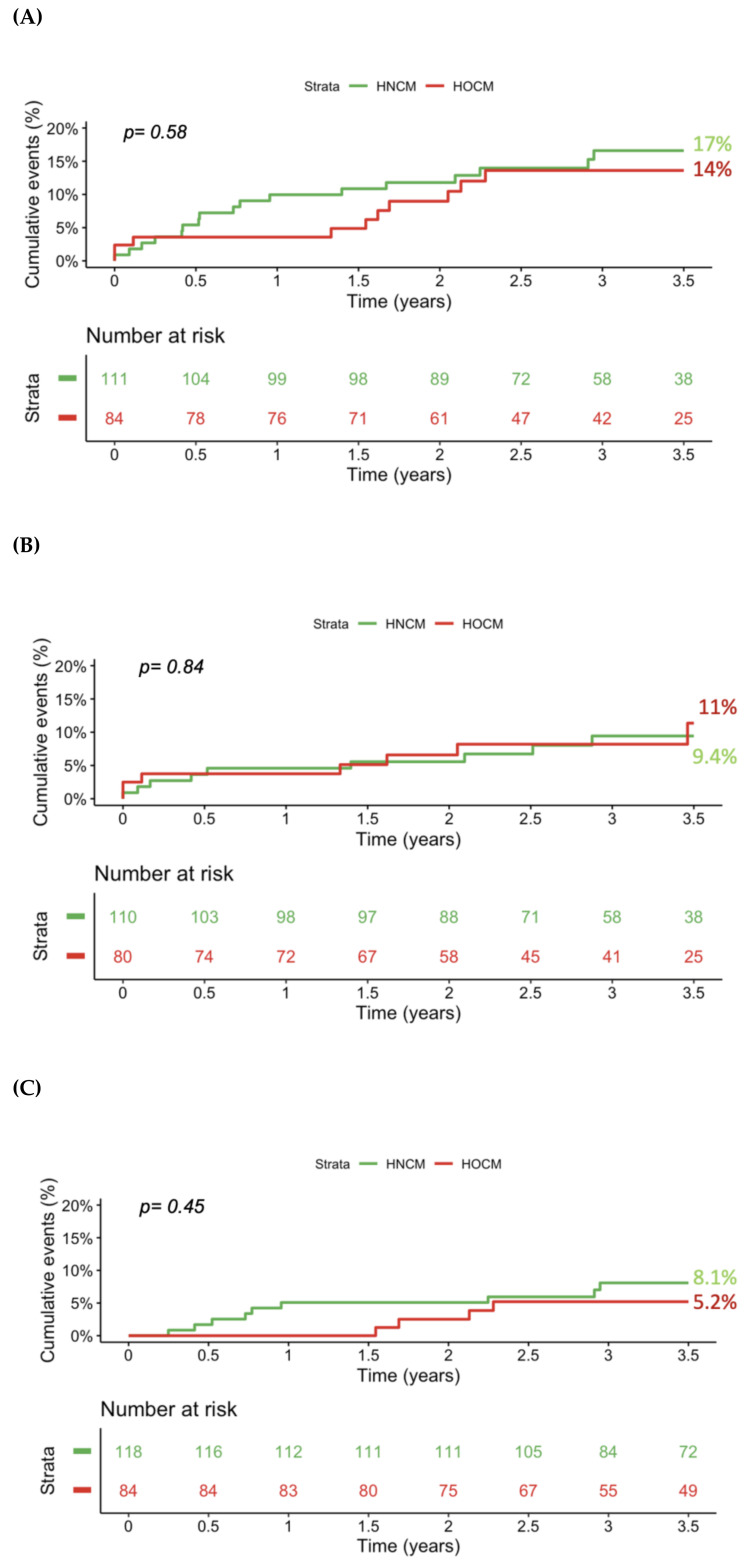
Kaplan–Meier curves and the corresponding event rates for (**A**) the composite of new-onset VT, appropriate ICD therapy, and all-cause mortality; (**B**) new-onset VT; and (**C**) appropriate ICD therapy and all-cause mortality stratified by the presence of significant LVOT obstruction. ICD, implantable cardioverter defibrillator; LVOT, left ventricular outflow tract; VT, ventricular tachycardia.

**Figure A2:**
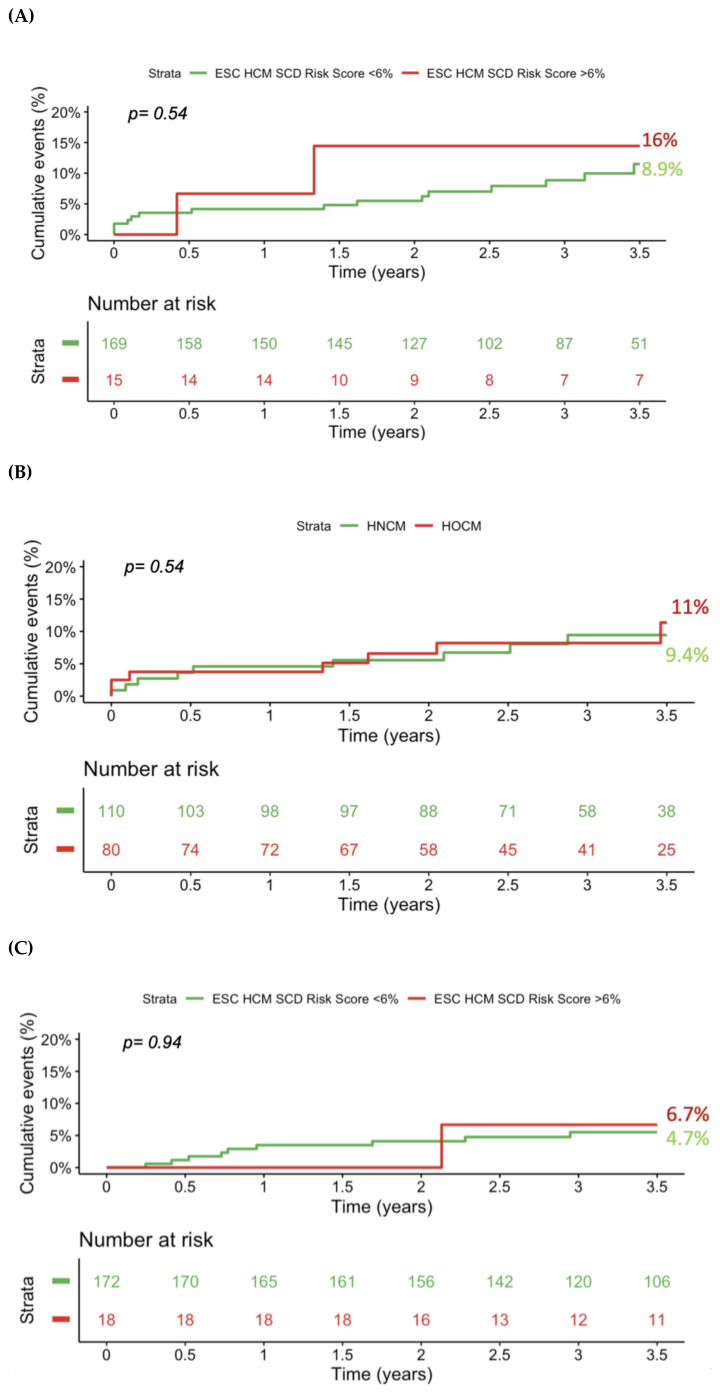
Kaplan–Meier curves and the corresponding event rates for (**A**) the composite of new-onset VT, appropriate ICD therapy, and all-cause mortality; (**B**) new-onset VT; (**C**) appropriate ICD therapy and all-cause mortality stratified by an ESC-HCM-SCD risk score cut-off of 6%. ICD, implantable cardioverter defibrillator; VT, ventricular tachycardia.

When analyzing different subgroups, our model demonstrated consistent effectiveness across sex, age above and below the median, and the presence of an LVOT obstruction, with no significant interaction effects (*p* for interaction = 0.37, 0.71, and 0.21, respectively). We were not able to show differences regarding clinical outcomes in patients with and without significant LVOT obstruction (Appendix Figure A1), and similar results were obtained in patients above and below a 5-year SCD risk of 6% derived by the risk calculator of the ESC (Appendix Figure A2). As no patient in the intermediate risk category (4%–6%) had LGE exceeding 15%, the 2023 guideline update did not alter risk classification in our cohort.

Furthermore, ROC analysis indicated a moderate discriminatory performance capacity for the percentage of LGE (AUC 0.72, *p* = 0.010). In addition, the Youden index revealed an optimal LGE cut-off of 5.25%. When adding a history of at least one syncope as a clinical risk factor to the model, the ROC curve increased significantly to 0.77 (Δ0.05; *p* = 0.020). In contrast, the AUC for the ESC-HCM risk score was 0.65, which was significantly lower compared with the AUC of LGE (*p* = 0.011) and the combined AUC of LGE + syncope (*p* = 0.001).

## 4. Discussion

HCM presents with diverse clinical manifestations across all age groups, making the prediction of SCD a critical challenge in patient management.

This study emphasizes the significance of LGE quantification on cardiac MRI and a history of unexplained syncope as essential factors for stratifying patients at risk of adverse outcomes. Our key findings may offer a new approach to refining risk stratification in HCM patients. 

Our chosen LGE cut-off diverges from the current clinical guidelines, which recommend a 15% threshold [3,14]. This value is notably high; in our cohort, only a few patients reached such an extent of LGE. Previous studies have also investigated a 10% cut-off as a risk predictor in patients with HCM [7,8]. However, our analysis and Greulich et al.’s findings suggest that a lower cut-off of 5% is more clinically relevant [9]. Greulich et al. studied 220 patients with baseline characteristics similar to those in our cohort regarding age, sex, and LGE extent, with a median follow-up of slightly more than 10 years. A key difference in their study was the lower incidence of unexplained syncope, which precluded the development of a combined risk model using imaging and clinical parameters. Our analysis further validated this 5% threshold using the ROC-derived Youden index, identifying an optimal cut-off of 5.25%.

After multivariable adjustment, the extent of LGE was independently associated with the combined endpoint. However, its association with the secondary endpoints of appropriate ICD therapy and all-cause mortality did not reach statistical significance, possibly due to the small sample size. However, we showed a strong association between LGE and newly-detected VTs, frequently preceding more severe arrhythmia [15]. We found that the presence of even short non-sustained VTs (nsVTs) may likewise be reliably predicted by LGE in HCM patients, and therefore, the extent of LGE could more commonly be used for risk prediction in the early stages of the disease. Given these insights, our results potentially raise a paradigm shift in clinical practice regarding monitoring patients exhibiting the aforementioned risk markers. Specifically, these observations may raise the question of whether affected patients warrant more vigilant surveillance, potentially necessitating shorter intervals of Holter-ECG assessments or the implantation of loop recorders due to regular underdiagnosis of nsVTs in solely one Holter examination per year. Noteworthy, our findings emphasize the importance of CMR imaging with LGE quantification at regular intervals, as currently suggested by the ESC guidelines [3].

Given the study's prospective design, we excluded patients with a previous history of VTs, leading to a lower proportion of individuals with an ESC-HCM-SCD risk score exceeding 6%. Notably, we did not observe significant disparities in event rates when comparing patients with risk scores above and below this threshold. Furthermore, the AUC of the ESC-HCM-SCD risk score was significantly lower than our proposed risk stratification model. These findings may challenge the sensitivity and specificity of the current risk score in identifying patients at high risk. 

The currently renewed cardiomyopathy guidelines of the ESC addressed some of these issues by refining their risk assessment strategy for HCM patients. They now recommend considering CMR parameters in patients with an intermediate ESC-HCM-SCD 5-year risk of 4–6%. This update emphasizes the role of advanced imaging techniques in improving predictive accuracy [3]. In addition, Wang et al.'s research further illuminates this area. Their findings of 774 patients suggest that while the 2023 update enhances the predictive power of the risk score, the utilized cut-off of the extent of LGE might be inadequate [16]. However, the aforementioned study addressed LVOT gradients only at rest, which could consequently lead to underestimating the ESC-SCD risk score.

Nevertheless, they propose 5% as the best threshold for LGE in risk prediction. This aligns with our results that lower thresholds of LGE combined with clinical aspects like syncopal episodes may significantly improve risk stratification. This pivotal insight from our analysis underscores the indispensable value of meticulous medical history taking. Each patient was evaluated at a tertiary referral center specialized for HCM, with pointed inquiries about syncopal episodes and their specific nature. This finding reaffirms that while advanced diagnostic modalities are integral, a thorough patient history remains crucial in clinical management, serving as a vital tool in risk assessment for HCM patients [17,18]. Similarly, Maron et al. strongly encourage primary-prophylactic ICD implantation in patients with at least one clinical risk factor, such as syncope [4].

In patients with an LGE extent below 5% and the absence of syncope, we did not observe any events regarding the secondary endpoint of appropriate ICD therapy and all-cause mortality, which highlights the potential of these markers in delineating a low-risk population in HCM. Overestimating SCD risk in this vulnerable patient population may lead to unnecessary device implantation, including potential side effects such as uncontrolled infection, inappropriate device therapy, or lead dislocation.

Moreover, as mentioned above, traditional risk stratification tools rely heavily on the dimension of measured LVOT gradients. Our findings, which revealed similar event rates in patients with non-obstructive HCM compared with the obstructive HCM cohort, highlight a significant limitation of this approach. Specifically, these models may underestimate the individual risk of patients with non-obstructive HCM, raising the imperative need for a more individualized risk assessment strategy in these individuals. In addition, our risk model showed that its predictive power does not change between obstructive and non-obstructive patients.

Mavacamten, a selective myosin inhibitor, has already demonstrated efficacy in alleviating the symptoms of obstructive HCM, predominantly by modulating LVOT gradients. While its capacity to reduce the LVOT gradient and, subsequently, the HCM-ESC-SCD risk score is recognized, its comprehensive impact, particularly concerning SCD prevention, remains to be determined. Mavacamten trials have shown a decrease in high-sensitive serum troponin levels after specific treatment periods, mitigating the drug’s potential to reduce cardiac stress and fibrosis. Nevertheless, the effects of long-term mavacamten therapy with respect to scar reduction and/or a decrease in unfavorable arrhythmic events remain to be evaluated in a larger patient population [19,20,21].

## 5. Limitations

Our study has several limitations. Given its observational design, the potential for unobserved confounders cannot be excluded. The specific characteristics of our cohort could prevent the extrapolation of our findings to a broader HCM demographic. The detection of our endpoints, particularly the novel onset of VTs, bears the challenge that only a minority of our participants had an implantable loop recorder or an ICD, and the majority underwent just one annual 48-hour Holter-ECG. Although we observed significant associations, there were few events in this analysis. Larger prospective studies are needed to validate these findings. In addition, the precise technique of LGE percentage measurement remains challenging, and other studies have used different approaches to quantify the LGE. We used the full width at half-maximum method, which is described to have a good intra- and interobserver variance. However, it may underestimate the amount of LGE [22]. Furthermore, we did not assess the spatial distribution of LGE.

## 6. Conclusions

In conclusion, our findings indicate that the combination of a history of syncope together with small amounts of LGE (≥ 5%) in cardiac MRI are associated with unfavorable clinical outcomes in HCM patients.

## Figures and Tables

**Table 1:** Baseline characteristics.

	Overall (*n* = 230)	No Event (*n* = 201)	Event (*n* = 29)	*p*-Value
**Clinical parameters**				
Age, years	56 (44, 64)	56 (43, 64)	57 (47, 64)	0.84
Sex male, *n* (%)	136 (60%)	118 (60%)	18 (62%)	0.83
HOCM, *n* (%)	84 (43%)	74 (44%)	10 (34%)	0.36
Body mass index, Kg/m^2^	28.7 (26.0, 31.9)	28.7 (25.8, 31.7)	28.7 (26.6, 32.0)	0.55
NYHA functional class ≥2, *n* (%)	143 (67%)	125 (68%)	18 (64%)	0.70
6-MWD, m	483 (410, 535)	486 (411, 558)	445 (396, 505)	0.29
ESC-SCD risk score, %	2.92 (2.14, 4.13)	2.76 (2.08, 3.96)	4.06 (2.95, 4.42)	0.14
Prior ICD implantation, *n* (%)	25 (11%)	22 (11%)	3 (10%)	0.93
History of syncope, *n* (%)	45 (20%)	32 (16%)	13 (46%)	**<0.001**
Coronary artery disease, *n* (%)	44 (20%)	37 (19%)	7 (24%)	0.57
Arterial hypertension, *n* (%)	124 (55%)	112 (57%)	12 (41%)	0.12
Diabetes mellitus type 2, *n* (%)	37 (17%)	33 (17%)	4 (14%)	0.81
Atrial fibrillation, *n* (%)	52 (23%)	44 (22%)	8 (28%)	0.52
Prior myectomy, *n* (%)	4 (1.7%)	3 (1.5%)	1 (3.4%)	0.28
Coronary artery disease, *n* (%)	44 (20%)	37 (19%)	7 (24%)	0.53
**Medication**				
Beta blocker, *n* (%)	148 (66%)	127 (65%)	21 (72%)	0.49
ACE inhibitors, *n* (%)	44 (20%)	39 (20%)	5 (17%)	0.70
Angiotensin receptor blocker, *n* (%)	66 (29%)	59 (30%)	7 (24%)	0.56
Alpha blocker, *n* (%)	23 (10%)	22 (11%)	1 (3.4%)	0.31
Diuretics, *n* (%)	29 (13%)	21 (11%)	8 (28%)	**0.032**
Anticoagulants, *n* (%)	36 (16%)	34 (18%)	2 (6.9%)	0.21
Antiplatelet agents, *n* (%)	60 (27%)	52 (27%)	8 (28%)	>0.9
**Laboratory parameters**				
Hb, g/dL	14.30 (13.13, 15.28)	14.30 (13.20, 15.30)	14.20 (12.70, 15.00)	0.50
Creatinine, mg/dL	0.91 (0.79, 1.07)	0.91 (0.78, 1.06)	0.94 (0.80, 1.21)	0.32
eGFR, ml/min/1.73 m^2^	80 (62, 97)	80 (62, 98)	81 (60, 94)	0.58
CRP, mg/dL	0.19 (0.09, 0.42)	0.18 (0.08, 0.38)	0.30 (0.18, 0.55)	0.22
hsTnT, ng/L	13 (9, 24)	12 (8, 20)	22 (13, 30)	0.080
NT-proBNP, pg/L	493 (164, 1230)	454 (145, 1215)	654 (430, 1295)	0.39

Continuous data are reported as median (interquartile range). 6-MWD, 6-min walk distance; ACE, angiotensin-converting enzyme; CRP, C-reactive protein; eGFR, estimated glomerular filtration rate; ESC-SCD risk score, European Society of Cardiology sudden cardiac death risk score; Hb, Hemoglobin; HOCM, hypertrophic obstructive cardiomyopathy; hsTnT, high-sensitivity troponin T; ICD, implantable cardioverter defibrillator; NT-proBNP, N-terminal pro-B-type natriuretic peptide; NYHA; New York heart association; bold: *p*-value below 5%.

**Table 2:** Imaging characteristics.

	Overall (*n* = 230)	No Event (*n* = 201)	Event (*n* = 29)	*p*-Value
LA volume index, cm^3^/m^2^	27.8 (24.1, 30.9)	27.6 (24.1, 30.9)	28.4 (24.0, 30.6)	0.74
LA, mm	58 (54, 64)	58 (54, 64)	58 (56, 65)	0.33
LA longitudinal strain, %	15 (9, 21)	15 (10, 21)	11 (7, 20)	0.22
RA volume index, cm^3^/m^2^	25.8 (23.3, 28.9)	25.5 (23.3, 28.9)	26.9 (23.4, 27.9)	0.66
RA, mm	50 (47, 55)	49 (47, 55)	53 (46, 58)	0.46
RV volume index, cm^3^/m^2^	15.24 (13.8, 17.1)	15.27 (13.8, 17.0)	14.74 (13.8, 17.2)	>0.9
RV ejection fraction, %	57 (51, 64)	57 (51, 64)	57 (50, 67)	0.90
Interventricular septum, mm	19.0 (17.0, 22.0)	19.0 (17.0, 22.0)	20.0 (17.0, 22.0)	0.90
LV volume index, cm^3^/m^2^	21.1 (18.9, 23.3)	21.1 (18.8, 23.3)	21.2 (19.7, 23.3)	0.72
LVOT gradient > 30 mmHg, *n* (%)	84 (43%)	74 (44%)	10 (34%)	0.36
LV ejection fraction, %	65 (59, 71)	66 (59, 71)	65 (55, 72)	>0.9
Global longitudinal strain, %	14.5 (12.3, 17.0)	14.5 (12.2, 17.0)	15.0 (12.4, 17.4)	0.57
LV stroke volume index, ml/m^2^	52 (47, 62)	53 (46, 62)	51 (48, 60)	0.59
Cardiac output index, L/m^2^	3.39 (2.89, 3.99)	3.38 (2.89, 4.00)	3.43 (2.94, 3.99)	0.61
Extra cellular volume, %	28.5 (26.5, 30.6)	28.2 (26.0, 30.0)	29.7 (27.6, 32.3)	0.052
Presence of LGE, *n* (%)	68 (54%)	53 (51%)	15 (71%)	0.078
Extent of LGE, %	1.8 (0.0, 5.4)	1.1 (0.0, 4.0)	9.0 (0.8, 13.2)	**0.012**

Continuous data are reported as median (interquartile range). LA, left atrium; LGE, late gadolinium enhancement; LV, left ventricle; LVOT, left ventricular outflow tract; RA, right atrium; RV, right ventricle; bold: *p*-value below 5%.

## Data Availability

The data underlying this article will be shared on reasonable request to the corresponding author.

## Abbreviations

The following abbreviations are used in this manuscript:

area under the curve	(AUC)
cardiac magnetic resonance imaging	(CMR)
electrocardiography	(ECG)
European Society of Cardiology	(ESC)
hypertrophic cardiomyopathy	(HCM)
implantable cardioverter defibrillator	(ICD)
Kaplan–Meier	(KM)
late gadolinium enhancement	(LGE)
left ventricle	(LV)
LV outflow tract	(LVOT)
non-sustained VTs	(nsVTs)
Receiver Operating Characteristic	(ROC)
sudden cardiac death	(SCD)
transthoracic echocardiography	(TTE)
ventricular tachycardia	(VT)
